# An Overview on Coronary Heart Disease (A Comparative Evaluation of Turkey and Europe) and Cost-effectiveness of Diagnostic Strategies

**DOI:** 10.4274/MIRT.33

**Published:** 2011-12-01

**Authors:** Cengiz Taşçı, Nihat Özçelik

**Affiliations:** 1 Gama Medical Center, Derpartment of Nuclear Medicine, Gaziantep, Turkey

**Keywords:** Coronary heart disease, cardiac noninvasive testing, cost-effectiveness, myocardial perfusion scan

## Abstract

**Objective:** Coronary heart disease (CHD) is the leading cause of death for men and women in Turkey as it is in Europe and US. The prevalence of the disease is 3.8% in Turkey and 200,000 patients are added to the pool of CHD annually Because of genetic predisposition and high proportions of physical inactivity, smoking habit, and obesity, CHD is encountered in earlier ages in our country So, the economic burden of the disease is expected to be relatively high, but the amount of health expenditure is not always parallel to the prevalence of a disease in the community. This article was written to overview CHD statistics to make a comparison between Turkey and some European countries and to investigate the value of myocardial perfusion scan (MPS) as a gatekeeper in diagnosing CHD before invasive coronary angiography (ICA). The consequences were evaluated for Turkey In diagnosis; noninvasive testing gains importance in connection with the new approaches in treatment strategies, because a direct ICA strategy results in higher rates of revascularization without improvement in clinical outcomes. A "gatekeeper" is needed to select the patients who are not required to undergo angiography. MPS with its proved power in diagnosis and predicting prognosis, provides a cost-effective solution, and is accepted in some extensive analyses as a "gatekeeper" particularly in intermediate and high risk patients and in patients with known CHD. In conclusion, MPS may provide an optimal solution better than the ongoing situation in Turkey as well, when it is approved as a "gatekeeper in an algorithm before ICA.

**Conflict of interest:**None declared.

## INTRODUCTION

**The Prevalence and the Incidence of CHD in Europe and Turkey**

**Figure 1:**
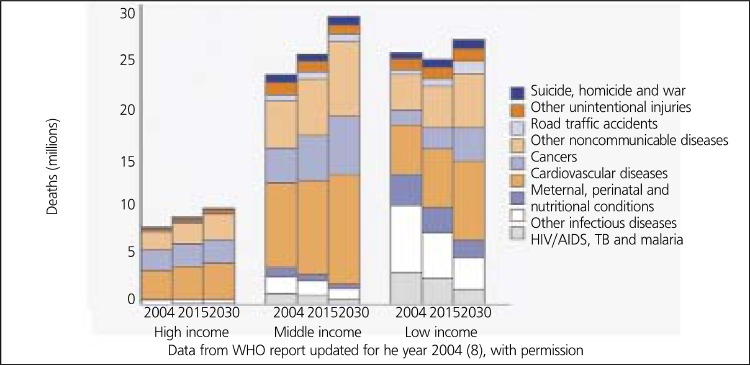
Deaths by cause for high-, middle and low-income countries in theFuture

Coronary heart disease (CHD) is an important health problem in Turkey as it is in Europe and US, because it is the leading cause of death for men and women, and it causes substantial disability and loss of productivity (1,2,3,4,5). The World Health Organization (WHO) assessments about 15 leading causes of death worldwide had indicated that ischemic heart disease would move from number five position in 1990 to number one position in 2020 (2,4). This estimation was first made in 1996, but the recent studies evaluating the global burden of disease (last updated for 2004) revealed that this had become already a fact particularly in the developed and developing countries (6,7,8). According to the WHO projections, the number of deaths due to CHD will increase in the future all over the world. This trend is expected to continue for the next 30 years (8) (Figure 1).

**Figure 2:**
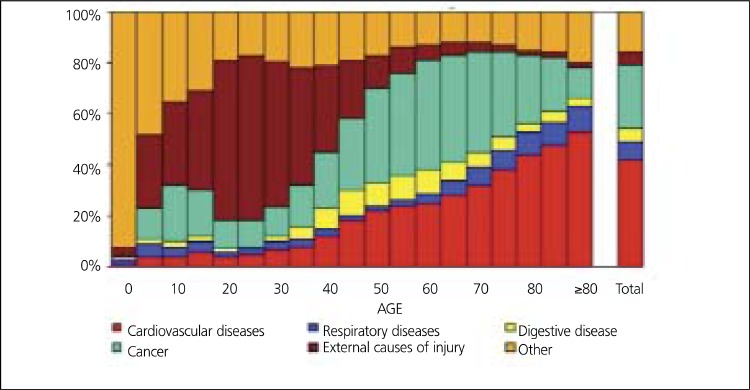
Major causes of death by age in Europe from Euro Heart survey2006 (9)

**Figure 3:**
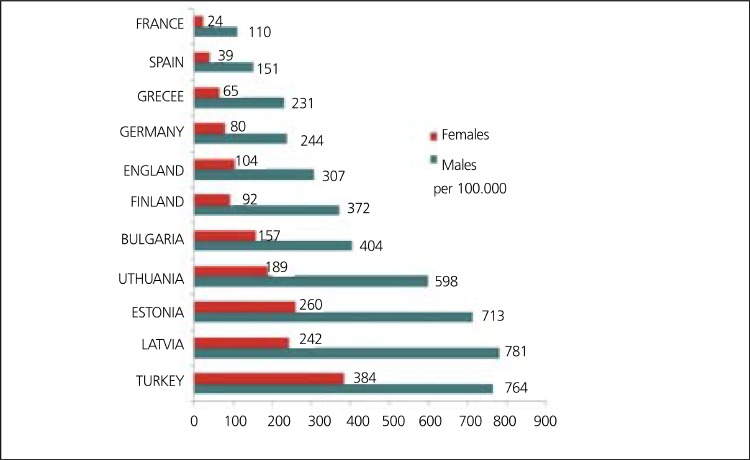
Age standardized mortality rates in 45-74 years old people withCHD in Europe, 2000 (15,16,17). Modified from Ref 15

European cardiovascular disease (CVD) statistics indicate that CHD by itself is the most common cause of death in Europe and in European Union (EU) (Figure 2). 1.92 million deaths (21% of men and 22% of women deaths) from CHD in Europe and 741,000 deaths (16% of men and 15% of women deaths) in EU are accounted each year There is a marked west-east gradient in the age standardized cardiovascular mortality rates. Death rate from CHD is relatively low and decreases steadily in Northern, Southern and Western Europe, while it is high and increases in Central and Eastern Europe including Turkey. Cardiovascular mortality rates for women are lower than those for men in all European countries (3,9,10,11,12) (Figure 3).

**Figure 4:**
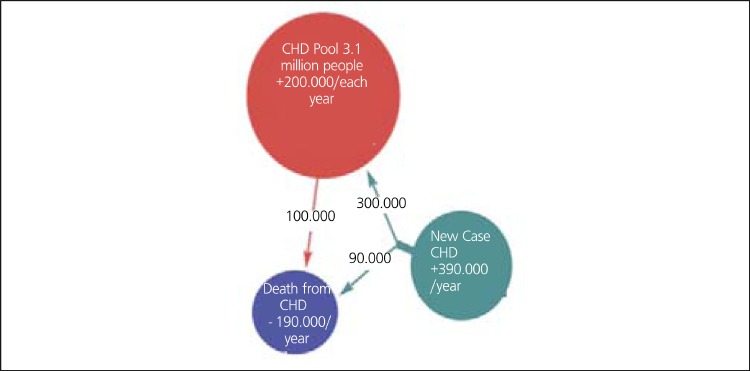
Patient population, new cases and death from CHD in Turkishadults ([ref:15]15[/ref]). With permission of A. Onat

"Turkey Burden of Disease Study, 2004" by Refik Saydam Hygiene Center (RSHC) on behalf of the Ministry of Health and TEKHARF Studies by A. Onat et al. also confirm that ischemic heart disease is the leading cause of death in Turkey (1,13,14,15) (Table 1). CHD is responsible for 20.7% of male, and 22.9% of female deaths (21.7% of all) (1). These numbers are very close to the average of Europe, but higher than the average of EU (3,9,10,11,12,15). As it is in the other Eastern European Countries, CHD rates increase each year in Turkey (13,14,15). The prevalence of the disease in individuals over 50 years old was found to be increased by 80% in 2007-2008 TEKHARF study, when compared to the study in 1990 (15). The total prevalence is 3.8% (4.1% in males and 3.5% in females). There were approximately 1 million patients in 1990 and the number reached 3.1 million up to 2008. 390 000 new coronary artery events and 190 000 deaths are encountered each year. This means that 200 000 patients are added to the pool of CHD yearly (15) (Figure 4). Coronary mortality rate in 45-74 year-old individuals is reported as 0.57% (0.76% in males and 0.38% in females) between 1990-2008 and a decreasing trend in rising rates was recorded between 2000-2008 (15,16).

In an European analysis in 2000 that did not include Russia and Ukraine, the annual mortality from CHD in 45-74 years of age was found highest in Turkish women and second highest in Turkish men after Latvians (Figure 3). Coronary mortality rates are approximately 3 times more in Turkish men and 5 times more in Turkish women than those in Western Europe (15,16,17).

CHD is encountered in earlier ages in Turkey. The prevalence of the disease is about 6% in 45-54 year-old individuals, which is considered to be relatively high for this age population (15). Every ten years of aging increases the risk of CHD 1.8 fold in men and 1.9 fold in women (18). Actually, Turkey is a unique country with its young population among European countries. Population over 65 years old (65+) was 5.8% in 2008, while the average of European countries was 15.3% (Table 2). Since the age is the most important independent risk factor (18) and CHD is seen generally in elder people over 65 years old (65+), it is surprising that CHD is the first cause of death in Turkey as it is in the developed countries with aged populations. 2009 statistics by Turkish Ministry of Health indicated that population aged 65+ was 4.3% in 1990, 5.7% in 2000, 6.7% in 2008 (not age-standardized), and 7.0% in 2009 (19). Very far off from the rates in Europe but the statistics indicate an increase in elder people in Turkish population probably because the average life and life expectancy is getting longer due to advanced treatment options. On the other hand, CHD is a complex disorder resulting from many risk factors. Genetic predisposition for atherosclerosis is a substantial risk for developing CHD especially at early ages. Turkish adults -both men and women- have the lowest levels of total cholesterol (TC) and HDL-C among the citizens of all European countries (Table 3). Several comparative studies including Turks living in Germany and US confirm that HDL-C levels in Turks are among the lowest in theworld. Therefore, the ratio of TC/HDL-C, the best independent lipid predictor of CHD, is very high in Turkish adults. Low levels of TC, LDL-C and HDL-C are associated with high levels of hepatic lipase, fasting triglycerides and high levels of apolipoprotein-B (20,21,22,23,24). This lipid profile pointing out a genetic disorder constitutes a remarkable early predisposition to CHD.

Positively, the percentage of total energy available from fat is relatively low in Turkish people and the average amount of fruit and vegetable intake per person is the second greatest in Turkey after Greece among all European countries (Table 4). There are some regional differences in diet habits ranging from the Aegean coast diet rich in olive oil to the inland Anatolian diet rich in meat and pastry. Alcohol consumption is distinctively low in our country, so that moderate alcohol consumption reduces the risk of CHD while high level of intake increases (Table 4). But, physical inactivity common in both genders, smoking habit especially in men and obesity highest in Turkish women in Europe result in HT, diabetes, metabolic syndrome and finally a high prevalence of CHD (Table 3). All these factors probably are the reasons of unanticipated fact that Turkish adults have the pattern of death causes similar to the population of developed countries (20). In paralel to these predispositions, TEKHARF studies indicate that Framingham risk scoring when applied to the Turkish adults underestimates the risks in reality, because the absolute coronary event risk is much higher in Turkey (18).

**Economic Burden of CHD**

**Figure 5:**
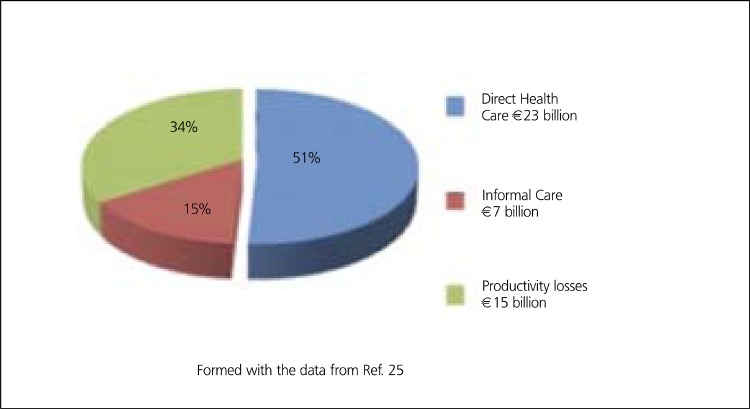
Economic burden of CHD in EU, in 2003 (Overall cost is 49 bilion a year)

**Figure 6:**
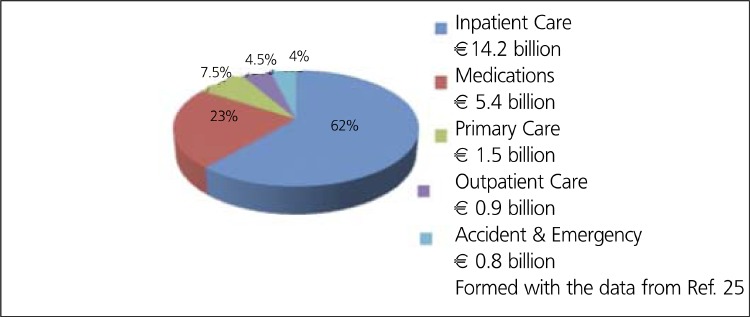
Direct health care cost for CHD in EU ( 23 billion a year)

CHD is estimated to cost the EU economy 49 billion a year, about 2.6% of total healthcare expenditure. CHD healthcare cost per capita is about 50 in EU, when purchasing power parity (PPP) is used (25) (Table 5). Of the total cost of CHD in EU, about 48% is due to direct health care costs, 34% to productivity losses and 18% to the informal care costs. Of the total direct healthcare costs, about 62% is due to inpatient care, 23% to medications, and remaining 16% to primary care, outpatient care and accident & emergency (25) (Figure 5,6).

**Figure 7:**
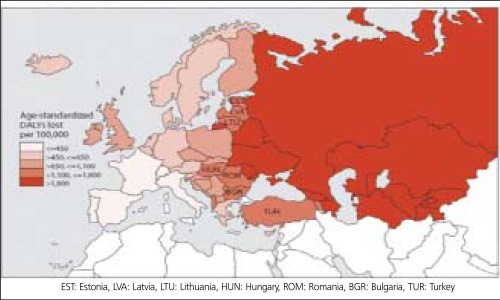
Age-standardized DALYs rate for CHD, 2002, Europe (11)

**Figure 8:**
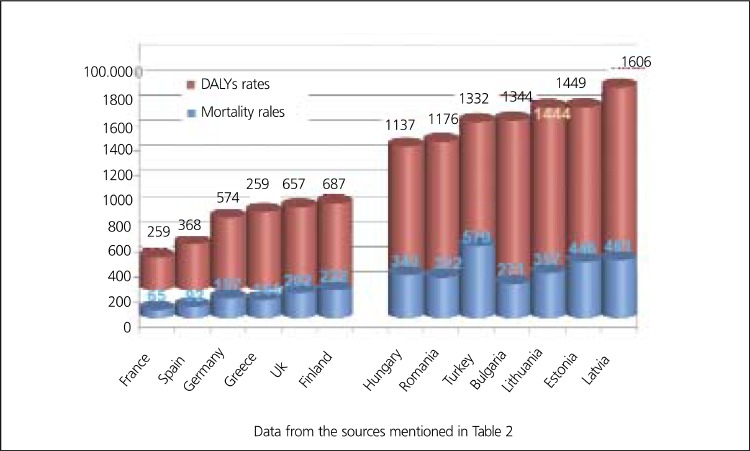
Age-standardized DALYs rates and mortality rates in someEuropean countries

**Figure 9:**
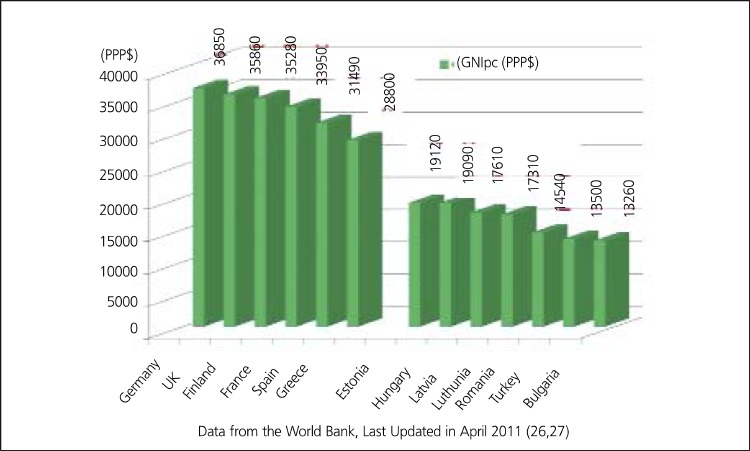
Gross National Income Per Capital (GNIpc) of the same Europeancountries (PPP$)

Unfortunately, there is no data available about disease- specific costs in Turkey, but some projections may be done for the economic burden of CHD using data from EU. According to an analysis of Europe in 2002 (12), Estonia, Latvia, Lithuania, Hungary, Romania and Bulgaria seem to be the closest countries to Turkey in terms of age-standardized disability-adjusted life years (DALYs) rate for CHD (Table 2, Figure 7,8). DALYs for a specific disease are calculated as the sum of the years of life lost due to premature mortality (YLL) and the years lost due to disability (YLD) (6). One DALY is defined as the loss of one year of equivalent full health. So, DALYs rate represents the consequences of morbidity and mortality from a disease together. Mortality rates from CHD are not always parallel to the DALYs rates, that's why not mortality rates alone but DALY's rates for CHD may be expected to have correlation with total or per capita health expenditure on CHD. As it is expected, J. Leal et al., found no direct correlation between CVD-related health expenditure and mortality rates or life expectancy (25). Anyway, coronary mortality rates are close in these seven low-income countries and the rates are higher than those in high-income countries in Europe (Table 2, Figure 8). Life expectancy, the second lowest in Turkish population after Ukrainians, is also similar in these seven countries. In the same study by J. Leal et al., a strong positive correlation was indicated between CVD- related health expenditure and national income. The Gross National Income per capita (GNIpc) and the total health expenditure per capita in these seven countries are close, too (Table 5, Figure 9). Hospital discharge from CHD is found uncorrelated to the other parameters (Table 2). Turkey seems to be in the same class with these six Eastern European countries in terms of GNIpc, total health expenditure per capita and age standardized DALYs rate for CHD. So, the average cost per capita on CHD may be expected to be similar in this group of countries and estimated about 20 (PPP) as the average of 4 of 7 countries calculated from the same study for the year 2003. (Table 5). This may roughly represent the average per person in Turkey, too.

**Diagnostic Strategies in Stable CHD in Connection with the Treatment Strategies**

**Understanding the Biology of CHD**

CHD is a general term for atherosclerosis in the coronary vessels and it appears in various stages. Fatty material and other substances form a plaque on the walls of the vessels.This chronic process narrows the coronary arteries which supply blood and oxygen to the heart muscle. The lack of oxygen causing some local changes results in myocardial ischemia presented with chest pain, myocardial infarction (MI) when a coronary artery is blocked totally and may perhaps lead to death. However CHD seems to be an obstructive disease of the main coronary vessels and the routine practice of treatment is generally based on this definition. There are some other factors influencing the clinical results like endothelial dysfunction in microvascular bed that is also linked to atherosclerosis but with no obstruction. Vasospastic angina, a hyper-contraction of smooth muscle of a coronary artery without plaque formation may lead to MI or sudden death (33). The research on syndrome X, microvascular angiopathy and slow coronary flow indicate that CHD symptoms may appear and stress-induced ischemia may be shown in some patients whose all major coronary vessels are proved completely open (34,35,36). Such patients with severe endothelial dysfunction in the absence of obstructive CHD have also been shown to have increased cardiac events (37). Slow coronary flow is a good example indicating the importance of function more than structure, so that, contrast agent in invasive coronary angiography (ICA) moves forward slowly in some patients with angina pectoris when comparedto normal individuals, although the patients have evidentlynormal coronary anatomy (38).

On the other hand, clinical importance of obstructive CHDis not predictable according to the degree of narrowing,because there is no direct relationship between the degree ofstenosis and cardiac events (39). Some compensatingmechanisms occur in low and high-degree of stenosis. In earlyatherosclerosis with less than 50% stenosis in the vessels,plaque development and intimal thickening increase the totalvessel area (expansive remodeling) to maintain lumen sizeand blood flow (40). Expansive remodeling despite its role to prevent ischemia is linked to plaque vulnerability and acute coronary syndromes (ACSs) like unstable angina, MI or sudden death. Over time, this positive remodeling becomes insufficient and is replaced with constrictive (or negative) remodeling. Constrictive remodeling is associated with much severe stenosis limiting blood flow and results in ischemia and stable angina presenting a relatively stable situation despite more progression in atherosclerotic pathway (40). Unfortunately, vulnerable plaques are generally asymptomatic, non-obstructive lesions that may rupture abruptly; therefore they are responsible for over 50% of cases of sudden death and acute MI (41). The content of vulnerable plaques is the reason for their unstable character. They are the soft plaques covered by a thin fibrous cap and include a large lipid core within a large amount of cholesterol esters and abundant macrophages indicating active inflammation. Severe stenotic plaques (narrowing >80% of the lumen) are more fibrotic and stable which are covered by a thick fibrous cap including less lipid core and macrophages, but more vessel smooth cells, collagen fibers and calcification (42). Both plaques usually exist together in a patient, and any imaging method even ICA, a gold standard in defining the degree of obstruction, tells us very little about which plaque may be responsible of future cardiac events (43,44). Finally in late atherosclerosis, chronic ischemia triggers new blood vessel growth to restore blood flow and oxygen supply to the affected areas like rendering a non-surgical natural by-pass (45). Because MI frequently develops from previously non-severe (<50%) lesions, artificial revascularization therapies targeting severe stenotic plaquesdo not help prevent the cardiac events when there arevulnerable plaques at the same time (46,47). Furthermore,some studies (CASS, ACME, AVERT, RITA-2, COURAGE andBARI 2D) comparing medical and surgical treatment stronglyemphasize that coronary revascularization beyond optimalmedical therapy may offer no substantial prognosticimprovement in stable patients (48) (Table 6). All theseconclusions indicate that CHD is not a simple disease ofnarrowed coronary arteries.

**Treatment Strategies**

Treatment strategies in managing stable CHD patients are controversial and still discussed in several studies (49,50,51,52,53,54,55,56,57,58,59,60,61,62,63,64,65,66,67,68,69,70,71). "Table 6" shows the results of comparisons of two main strategies (medical therapy versus revascularization) and two revascularization techniques including PCI and CABG. In summary, angina relief in short term with both revascularization techniques is superior to medical therapy, but the positive effect size is becoming less important in long term. CABG is the most effective therapy for eliminating the anginal symptoms due to providing more prompt revascularization than with PCI. The need for additional intervention is higher in PCI patients than in CABG group, and the revascularization rates in PCI patients are comparable with the patients receiving medical therapy. Drug- eluting PCI seems superior to bare metal stenting in reducing restenosis but not death or MI. In addition, drug-eluting stents increase late stent thrombosis, and long term dual antiplatelet treatment is required. Revascularization therapies that have more procedural complications including death are shown to be beneficial in patients with proven large area of ischemia (>10%), uncontrolled or worsening angina despite optimal medical therapy, impaired left ventricle (LV) function, significant proximal left anterior descending (LAD) or left main coronary artery (LMCA) stenosis (>50%) and extensive multivessel disease. As the most important result pointing out function more than anatomy, revascularization with PCI or CABG has no significant additional effect on mortality and cardiovascular event rates (MI or stroke) when compared to optimal medical therapy (OMT) alone. So, the authors suggest that medical therapy should be the first-line strategy in stable CHD patients and revascularization can safely be deferred until anginal symptoms worsen to a point that invasive treatment is required. Naturally, the revascularization guidelines were updated to emphasize the need for an objective evidence of large area of ischemia as Class I-A recommendation for invasive treatment in stable patients (72,73,74). Diagnostic strategies were also influenced by these conclusions. Noninvasive testing against and with ICA gained a very important role to select the patients who benefit from revascularization.

**Diagnostic Strategies**

There are a lot of diagnostic tools to investigate CHD in patients with chronic stable angina, like patient history and laboratory tests revealing the cardiac risk of the individuals, electrocardiography (ECG), chest X-ray, exercise ECG, echocardiography, coronary computed tomographic angiography (CTA), coronary artery calcium (CAC) scoring, intravascular ultrasound (IVUS), cardiac magnetic resonance imaging (MRI), stress imaging with single photon emission computed tomography (SPECT) myocardial perfusion scan (MPS) or echocardiography, and finally ICA (Only MPS will be used for SPECT MPS, because planary MPS is not in use anymore). New hybrid devices and new multimodality noninvasive imaging techniques searching different features of the disease in different stages are announced each year, and these developments bring new discussions on management of CHD (75). Noninvasive testing in stable CHD patients is still one of the most argued issues in medicine, although the clinical management of patients is carefully outlined in the "Guidelines of American ColleEge of Cardiology (ACC)/American Heart Association (AHA)/American College of Physicians/ASIM for the Management of Patients with Chronic Stable Angina", that was first published in 1999, and updated in 2002, and "Guidelines on the management of stable angina pectoris" of European Society of Cardiology (ESC), in 2006" (76,77). These guidelines tabulate the multiple published data on diagnostic use of the tests, emphasize the evidence levels, appropriateness criteria or contraindications and establish some flow diagrams about initial clinical assessment, diagnosis and treatment. These guidelines particularly ACC/AHA guidelines designate the noninvasive tests (stress ECG, stress MPS, stress echocardiography) in a concept of "stress testing with or without imaging". Both guidelines place ICA as an invasive test at the end of the diagnostic flow diagram to be reserved particularly for the high risk patients who have severe or uncontrolled angina or an evidence of ischemia in the absence of disabling symptoms. So, the major purpose of the use of "stress testing with or without imaging" is to indicate an objective evidence of ischemia. Because ischemia means risk in CHD patients, these tests are valuable not only for demonstrating the disease, but also for risk stratification that has long been recognized as critical in the clinical management of stable patients. "Stress testing with or without imaging" can distinguish high-risk patients who may benefit from early ICA, from non-high risk patients in whom optimal medical therapy is enough to control the disease. The patients at intermediate pretest risk are supposed to get maximum benefit from a noninvasive test, because the test makes a real change in posttest probability in this group of patients. Relatively fewer ones are supposed to be seperated as at high risk after noninvasive testing who need further investigation and/or revascularization that are costly. The annual cardiac event rate in patients who are found to be at low risk by "stress testing with or without imaging" is less than 1% (which is similar for low risk Duke treadmill scores and normal studies of stress MPS or stress echocardiography) (78). So, the non-high risk patients will only be investigated further if their symptoms cannot be controlled with medical therapy alone. That's why ischemia searching strategy provides better prognostic outcomes with less expenditure while stenosis searching strategy causes unnecessary revascularization without any improvement in prognosis. Another potential advantage of noninvasive stress testing is the demonstration of ischemia in patients without obstructive CHD who do not need revascularization, although they have relatively poor prognosis (78).

In the routine practice, clinical presentation, severity of angina, pretest probability, expected clinical utility, economic availability, contraindications and patient preferences are considered for choosing the optimal diagnostic test or strategy. Hovewer, the appropriate use of the tests are established in the guidelines basically according to the existence of the symptoms and pretest probability. Diagnostic strategies for ACSs are beyond the scope of this article, and only the guidelines about symptomatic stable patients and asymptomatic adults considering "stress testing with or without imaging" will be mentioned here shortly. In asymptomatic adults (79), global risk scoring (such as the Framingham risk scoring) is recommended for cardiovascular risk assesment, but exercise ECG (that may only be considered for the sedentary adults before starting a vigorous exercise program), echocardiography, stress echocardiography and stress MPS are not indicated in low and intermediate risk asymptomatic patients. Stress MPS is stated to be considered for the advanced cardiovascular risk stratification only in high risk asymptomatic patients with DM or evident family history of CHD or previous risk assesment testing indicates high risk of CHD such as coronary calcium (CAC) score of >400. But, it is strongly emphasized that stress imaging tests should be reserved for the advanced cardiovascular risk stratification of the symptomatic patients. CTA is not indicated for cardiovascular risk assesment in asymptomatic patients (79).

In patients with stable angina, stress ECG without imaging is recommended for those with intermediate pre-test probability of CHD based on age, gender, and symptoms, if not unable to exercise or ECG displays nonspecific changes (76,77). For risk stratification, it is indicated for the patients undergoing initial evaluation and for those with known CHD who suffer from significant deterioration in symptoms but not after recent revascularization (76,77). Routine periodic testing once angina is controlled is not indicated (77). Stress testing with imaging (exercise or pharmacological stress echocardiography/MPS), as opposed to exercise ECG, is recommended for the following conditions: 1) complete left bundle-branch block, electronically paced ventricular rhythm, pre-excitation (Wolff-Parkinson-White) syndrome, and other similar ECG conduction abnormalities; 2) patients who have more than 1 mm of ST-segment depression at rest, including those with left ventricular hypertrophy or taking drugs such as digitalis; 3) patients who are unable to exercise to a level high enough to give meaningful results on routine stress ECG who should be considered for pharmacologic stress imaging tests; and 4) patients with angina who have undergone prior revascularization, in whom localization of ischemia, establishing the functional significance of lesions, and demonstrating myocardial viability are important considerations (76). Stress imaging methods are recommended for risk stratification to identify the extent, severity, and location of ischemia, to assess the functional significance of coronary lesions in planning PCI, to indicate the functional severity of intermediate lesions found in ICA, and to predict the outcome of the treatment strategies (76,77,80). MPS is also indicated in symptomatic patients with known CHD (76,77). Assessment of response to therapy is another goal of using MPS (80). Table 7, 8 and 9 summarize the characteristics of the tests used for showing stress induced ischemia.

Cardiac MRI, IVUS, SPECT/CT, PET/CT, PET/MRI and some other techniques are investigated for the diagnosis of CHD, but CTA is the most promising new diagnostic tool currently discussed as an anatomic but noninvasive test against the "stress testing with or without imaging". It is particularly proposed for the patients with more atypical symptoms and a lower likelihood of CHD (85). Some studies suggest that CTA may potentially reduce both the time spent and overall cost in the lower risk patients who otherwise would have been subjected to more expensive and possibly less accurate testing strategies (85). Shaw and Narula propose CTA for lower range of intermediate risk (<50%) patients as a first line noninvasive imaging test while they suggest MPS or PET for upper range of intermediate (50-85%) and high risk (>85%) patients based on consideration of added clinical value and economic outcomes(86). In a recent study with referral and some verification biases, Weustink et al. propose CTA for the intermediate risk patients (87). But, Gibbons strongly emphasizes that CTA has a fairly limited evidence base while "stess testing with or without imaging" has robust evidences from the randomized studies with huge populations (78). He also emphasizes the relationship between the current healthcare crisis in US and the usage of newly developing tests outside the guidelines without sufficient evidence (78). Although CTA is proved to detect coronary stenosis accurately, the clinical utility of the proved stenosis remains unclear, because CTA is a poor predictor of inducible ischemia not only in patients with <50% narrowing in a coronary vessel but also with >50% coronary stenosis. Furthermore, Gibbons points out that an anatomic approach with CTA may possibly reduce the life style and risk factor modifications in patients who are found to be normal or near-normal in the test. On the other hand, Min and Shaw, as it is written in a letter to editor for the same article, believe that "identification of individuals with less severe forms of atherosclerosis permits more aggressive risk factor modification and medical treatment at an earlier stage" (78). But, no data is available to support any of these ideas in terms of clinical utility and economic consequences. Table 10 shows the advantages and disadvantages of CTA as a cardiac noninvasive test.

Anatomic or functional approaches, in other terms, stenosis or ischemia searching strategies are in competition for the diagnosis of CHD, although in fact they are complementary for a more precise diagnosis in some situations. ICA as a gold standart in the evaluation of stenosis provides direct radiographic visualization of the structural features of the coronary artery lumen (85). MPS as a functional test is proved to be the most important tool to indicate ischemia, its region and severity and to predict prognosis which is more practical than stress echocardiography in outpatient clinics (76,78,86). To compare these two anatomic and functional approaches and express the picture particularly in terms of nuclear medicine, we can make a simple simile for the heart as a cultivated field and coronary vessels as the pipes carrying water. To evaluate the irrigation condition in a field, one tries to control each of the pipes and if something is found narrowing or blocking inside, that pipe will be opened if possible on site. Another option is looking at the field from above. Brown or black areas are interpreted as the lack of irrigation, yellow areas as poor perfusion and the green areas as normal situation. First option like ICA evaluates the pipes but is blind to the field and the second option like MPS evaluates the irrigation condition in the field but is blind to the pipes. The heart is so distinctive that reporting a coronary artery as completely open or blocked does not say anything about the perfusion in the field in some situations, because the heart has some renewing mechanisms itself by angiogenesis that result in new and rich collateral vessels carrying blood and oxygen to the end point and prevent the heart from ischemia even if a main vessel is completely blocked. On the other hand, CHD symptoms may appear when coronary anatomy is completely normal as it is mentioned before. So, if we want to understand what is happening in the field and predict response to therapy, we have to observe the perfusion and function of the myocardium directly or prove ischemia by the way of other stress testing methods indirectly. MPS does not measure the degree of coronary stenosis but detects the results of atherosclerotic disease as the abnormalities in perfusion and function of the myocardium related to the severity of stenosis. Collateral flow and underlying endothelial dysfunction are also important parameters effecting MPS results, which make us understand the whole picture about prognosis. In the future, an imaging tool uniting these anatomic and functional points of view or some hybrid systems supplying anatomic, functional and/or perfusion data at the same time may be developed, but currently, these data are basically obtained from different imaging methods. So, MPS is an indispensable test yet for not only determining the perfusion with function but also for its proved strength to predict prognosis particularly in intermediate and high risk patients.

**Figure 10:**
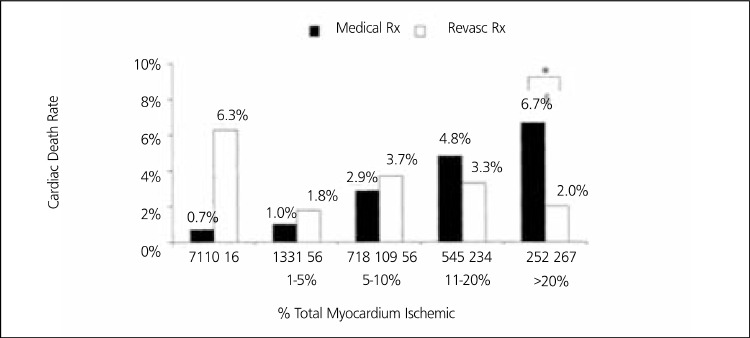
Observed cardiac death rates over the follow-up period in patientsundergoing revascularization (Revasc Rx) vs medical therapy (Medical Rx) as afunction of the amount of inducible ischemia. (P<0.0001) Ref. 88

All tests have some advantages and disadvantages, but the tests, in relation to treatment strategies, are expected to answer the question about what will happen to the patient. Will the test results change the way of therapy (surgical or medical) or will the patient benefit from the therapy which the test results point out. The underlying burden of atherosclerotic disease is often more severe than the burden of myocardial ischemia (86) but mostly, the severity of myocardial ischemia predicts prognosis and response to revascularization (68,86). So, risk determination in most patients is more valuable than defining the burden of atherosclerotic disease in deciding treatment strategy. MPS has a unique role in risk stratification and patient selection for revascularization by defining the extent and severity of ischemic myocardium. In a study by Hachamovitch (88), cardiac death rate was found to be directly associated with the relevant treatment strategy guided by the the severity of ischemia with MPS. Cardiac death rate decreases when the patients with moderate to severe myocardial ischemia undergo revascularization and the medical therapy is chosen in the absence of ischemia. Mortality increases if the patients with no myocardial ischemia undergo revascularization or those with moderate (5-10%) to severe (>10%) myocardial ischemia are medically treated (Figure 10). COURAGE nuclear substudy (68), a more recent randomized multicenter trial searching the value of MPS ischemia to guide therapeutic decision making, concluded that the addition of PCI to OMT resulted in more effective reduction of ischemia than OMT alone and complete normalization of MPS was more common in PCI+OMT group. Reductions in ischemia were associated with improvements in angina frequency and stability and >5% ischemia reduction was found related to considerably decreased cardiac event rate (68). Thus, a failure in reducing a patient's ischemic burden signifying a high-risk status warranted intensification of OMT and consideration of repeat angiography and/or revascularization (86). The magnitude of residual ischemia in follow-up MPS was proportional to the risk of cardiac events after either OMT or revascularization. More severe residual ischemia was associated with higher death or MI rates (68,86). So, despite its accuracy problems to indicate the atherosclerotic burden, MPS is the most powerfull independent predictor of prognosis (53,54,68,82,89,90). Anatomic approaches can detect the burden of atherosclerotic disease, but ischemia searching test is needed to see the light in the tunnel when considering the treatment strategies particularly in intermediate risk patients. CAC scoring (91,92,93) and CTA may be added to MPS (93,94,95) for a more complete evaluation in the conditions of unclear positive test results. SPECT/CT, PET/CT (96) or PET/MRI systems may be improved in the future to obtain the data of all aspects of coronary morphology with function for routine use.

Resource expenditure gains importance in noninvasive imaging of CHD, because of the various newly developed diagnostic techniques. Cost-effectiveness of the tests was searched comparatively in randomized and observational studies (Table 11). The diagnostic strategy of direct ICA in stable patients results in higher rates of coronary revascularization without improvements in clinical outcomes (82,86,97,98,99,100). That's why the seeking for a gatekeeper to select the patients who do not need to undergo ICA is put on the agenda. In most of the studies, MPS as a first line strategy is found to be highly cost-effective with its power in risk stratification when compared to ICA first strategy (82,86,97,98,99,100,101,102). Negative MPS results favorably exclude significant CHD and patients with normal MPS have <1% cardiac event rate in 2-3 years. Only 1% of the patients with normal MPS undergo downstream ICA (100). In an analysis based on the summary of the literature (100), 3 studies note that a strategy of "direct ICA" resulted in revascularization rates of 16% to 44% compared with the rates of 6% to 20% for "MPS first and then selective ICA" strategy without a negative impact on outcomes. In the END trial with 11,372 patients (97), revascularization rate was 73% in direct ICA strategy (3,958/5,423 patients) while it was 14.6% in "MPS first and then selective ICA" strategy (851/5,826 patients). In the second group, positive MPS rate was 34%. In other words, 2/3 of the patients were found to have normal MPS and excluded from additional expensive diagnostic evaluation. Diagnostic and 3-year follow-up costs were evidently high in patients undergoing direct ICA, because this strategy leaded more revascularization unrelated to the rate of CHD in the population. Composite cost of care was 30-41% lower in patients undergoing initial MPS. More ICA does not decrease the death or MI rates as it is believed and performed in the routine practice, because aggressive treatment may not always be considered appropriate, or result in improved cardiac outcomes. The EMPIRE study (82), a randomized multicenter study from Europe, proved that MPS using strategies were more cost-effective than strategies without using MPS, even than the initial stress ECG strategy. In fact, stress ECG without an imaging modality is the cheapest and widely available tool for evaluating the stress induced ischemia in patients with intermediate pretest probability, but its accuracy is low, that's why the overall cost of first-line strategy with this test is found to be higher than with MPS. A lower cost of a test alone does not necessarily result in a lower overall cost of patient care, because the cost of additional testing and intervention may be higher when the first-line test is less accurate (76,107).

Stress echocardiography is one of the cost-effective methods in competition with MPS in intermediate risk patients, but in a summary of the literature about cost- effectiveness of MPS revealed that 7 of 10 studies favored MPS for its technical advantages and well established pathway to ICA with comparable cost-effectiveness in long term (100). In a study by Shaw et al., stress echocardiography was found preferable in patients with low-to-intermediate pretest risk, while MPS was favorable in patients with intermediate-to-high pretest risk or known CHD. When compared to stress echocardiography, MPS was associated with early referral to angiography and revascularization (p<0.0001), and this resulted in a 3-year improvement in life expectancy (103).

In studies investigating the cost-effectiveness of CTA, for patients with suspected CHD, the high negative predictive value of CTA resulted in more cost savings when compared to MPS, and 9-month clinical outcomes were similar with these two modalities. But for patients with known CHD, cost of care was much higher with CTA, because of repeated ICA after the test (86,104,105). An anatomic diagnostic approach with CTA results in lower revascularization rates than with ICA, but, yet its percentage of PCI or CABG is 2-fold higher than with MPS. It appears that anatomic approaches, either invasive or noninvasive, result in higher rates of revascularization (86).

So MPS, as an outstanding noninvasive cardiac test providing a cost-effective solution besides its diagnostic and prognostic power, is accepted in some extensive analyses as a "gatekeeper" before ICA, particularly in intermediate and high risk patients and in patients with known CHD (82,97,100,102,106). In symptomatic patients with low pretest probability, MPS is indicated after equivocal or positive ECG stress test results or to clarify the significance of stenosis found in CTA when the results of these tests are not indicative for leading directly to ICA (76,86,107).

It may be thought that the results of the studies investigating the cost-effectiveness of noninvasive testing in US and EU may not reflect the economic outcomes in Turkey, because the prices for the tests are lower in our country. Some studies based on the diagnostic strategies should be planned for realizing the economic models in Turkey, but the comparative analysis based on PPP$ is expected to give similar results, because all the tests and the other services are cheaper in our country and furthermore the cost of a test alone does not have a great value in cost-effectiveness analysis of overall patient care.

As a provider and inspector of direct health care expenditure on behalf of the goverment, SGK, Turkish national social security establisment, performs package pricing for ICA. ICA and some PCI applications are evaluated in a package price. It seems logical at first glance, but is criticized in a study by Yılmaz MB et al. and they concluded that a rational cost-assessment system should take risk factors into consideration (108). They warn about the potential ethical problems particularly when the risky and complicated patients are the subjects. As it is extensively discussed here in this article, taking risk factors into consideration is much more important in noninvasive testing. Keeping MPS outside the package pricing is reasonable, because, in a given amount of money, a cheaper probably ineffective diagnostic test chosen for inappropriate population may lead to a more expensive overall patient care with an unnecessary invasive treatment. Another nonethical situation may appear when not using a noninvasive test under the pressure of economic limitations. The results of performing direct ICA are mentioned above in detail. In a study with 499 patients who underwent ICA in Israel, 58% of the procedures were found inappropriate due to error in management before performing ICA (109). Therefore, the test should be chosen in connection with the possible treatment strategies considering its power of risk determination. The pretest risk of the patients, the abilities of the individuals and their general health conditions should also be considered. Laupacis and colleagues have proposed guidelines for optimal integration of clinical and economic outcomes (110). Gibbons supports the guidelines as a solution for health care crisis in US until reaching a consensus on a new test or technique with reasonable evidences (78). Within the guidelines, some algorithms may be accepted for decision making. In Turkey as a middle-low income country, we'd better obey the guidelines for a sustainable health care system with little exceptions of some unsuitable conditions of the patients. MPS with robust evidences from the literature may provide not a perfect but an optimal solution when it is approved as a "gatekeeper in an algorithm before ICA. "MPS first and then ICA" strategy may provide better clinical and economic outcomes than ongoing situation in Turkey as well. Significant cost savings may be obtained without effecting clinical outcomes (97,100). There are currently sufficient number of nuclear medicine physicians and centers to realize this in our country (111).

Registration may guide the health policy makers to see the whole picture, but as it is emphasized in EUROASPIRE III study(112) recording patient data is still a problem in Turkey. Another problem is reaching the existing data. Disease specific data should be collected and shared with the professionals to foresee the clinical and economic consequences and plan the future.

As the MPS interpretors, nuclear medicine physicians should plan some studies not only focusing on the technical aspects of MPS but also its clinical usage, availability, clinical and economic outcomes, decision tree models, determining the indication, expectations from the test, clinician's perspective, patient's perspective, dealing with some spesific patients (women, diabetics, emergency department patients), improving and standardizing the technique, minimizing radiation exposure, standard reporting, etc.. All items need to be evaluated carefully. A recent study with striking results by Yapıcı O. indicates the need for clarifying the clinical usage of MPS in patients with different pretest probabilities (111). Some others should be expected.

**Primary Prevention vs Secondary Prevention **

Between 1981 and 2000, age specific CHD mortality in England and Wales fell by 62% in men and 45% in women aged 25-84. Approximately 58% of the fall in mortality was attributable to risk factor reductions —mainly smoking, cholesterol, and blood pressure (113). A study by Unal B et al. revealed that primary prevention had a fourfold greater impact than secondary prevention (113). Although the Turkish "prevention and control program for cardiovascular diseases" was first declared in 2007 (13,14) and the Turkish arm of EUROASPIRE study in 2010 concluded that the efforts for CVD prevention fall short of the targets similar to Europe (112), the efforts of struggling with smoking, obesity and HT should be maintained intensely. Such efforts are expected to reach their goal in long term. Thus, the prevalence of CHD will increase in the community in relation to increase in life in the future, and probably a greater attention to the issue will be needed.

**Abbreviations and Acronyms**

**ACIP:** Asymptomatic Cardiac Ischemia Pilot study

**ACME:** Angioplasty Compared to Medicine

**ARTS:** Arterial Revascularization Therapies Study

**AVERT:** Atorvastatin Versus Revascularization TreatmentBARI 

**2D:** Type 2 Diabetes and Coronary Artery Disease

**CABG:** Coronary Artery By-pass Grafting

**CASS:** Coronary Artery Surgery Study

**CECaT:** Cost-Effectiveness of functional Cardiac Testing.

**CCS:** Canadian Cardiovascular Society

**COURAGE:** Clinical Outcomes Utilizing Revascularizationand Aggressive Drug Evaluation.

**DM:** Diabetes MellitusEMPIRE: Economics of Myocardial Perfusion Imaging in Europe.

**END:** Economics of Noninvasive Diagnosis 

**HDL-C:** High density lipoprotein cholesterol 

**HT:** HypertensionLDL-C: Low-density lipoprotein cholesterol 

**MASS:** Medicine, Angioplasty or Surgery Study 

**MPS:** Single photon emission computed tomography, myocardial perfusion scan

**PPP:** Purchasing Power Parity. Exchange rate equalises the purchasing power of different currencies often used to compare the standards of living between countries.

**PCI:** Percutaneous coronary intervention 

**PTCA:** Percutaneous transluminal coronary angioplasty

**QALY:** Quality Adjusted Life Year.

**RITA-2:** Second Randomized Intervention Treatment of Angina

**SoS:** Surgery or Stent 

**TEKHARF:** Turkish Adult Risk Factor

## Figures and Tables

**Table 1:**
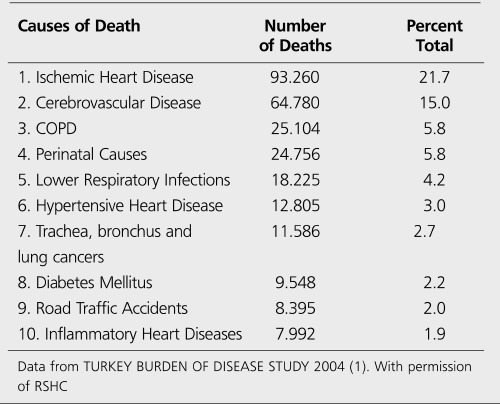
Ten Leading Causes of Death (Turkey, 2004)

**Table 10:**
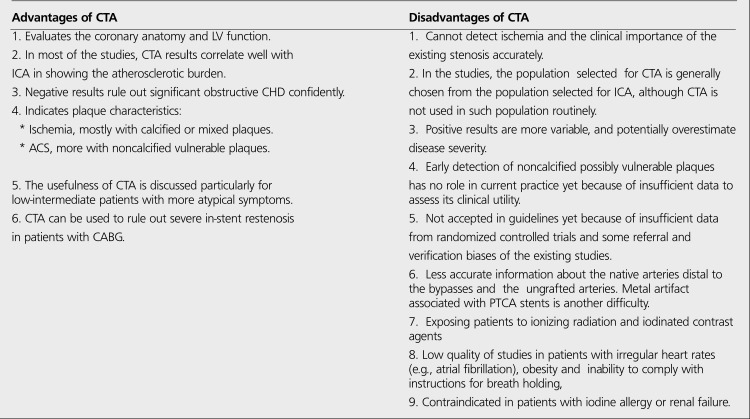
Advantages and disadvantages of CTA used in the diagnosis of CHD (78,85,86,87)

**Table 11:**
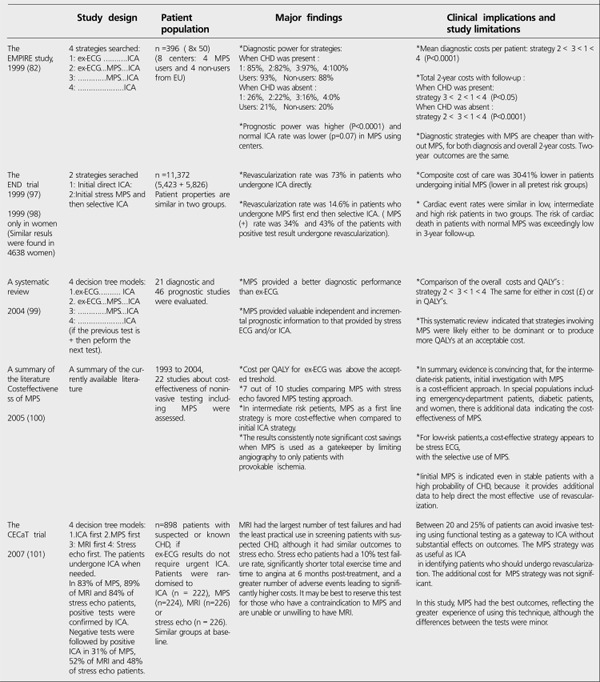
Cost-effectiveness trials based on the diagnostic strategies in stable patients with CHD

**Table 2:**
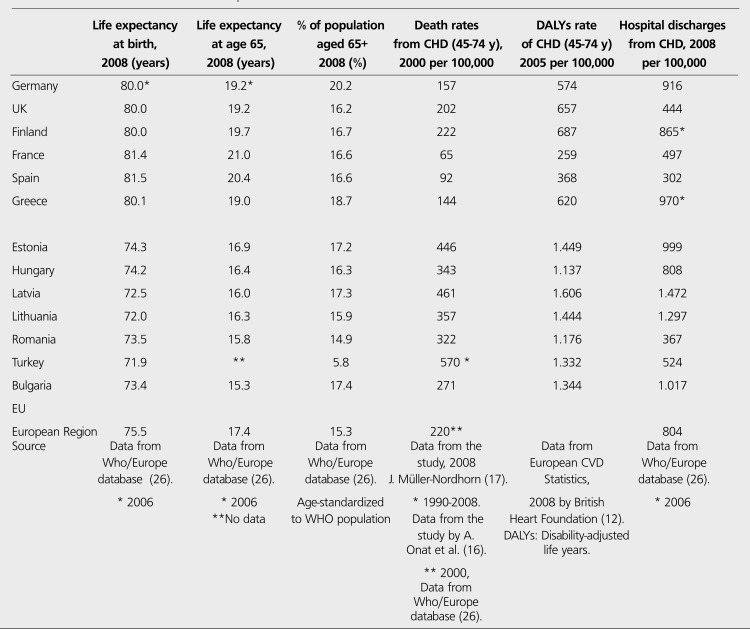
CHD related statistics in some European countries

**Table 3:**
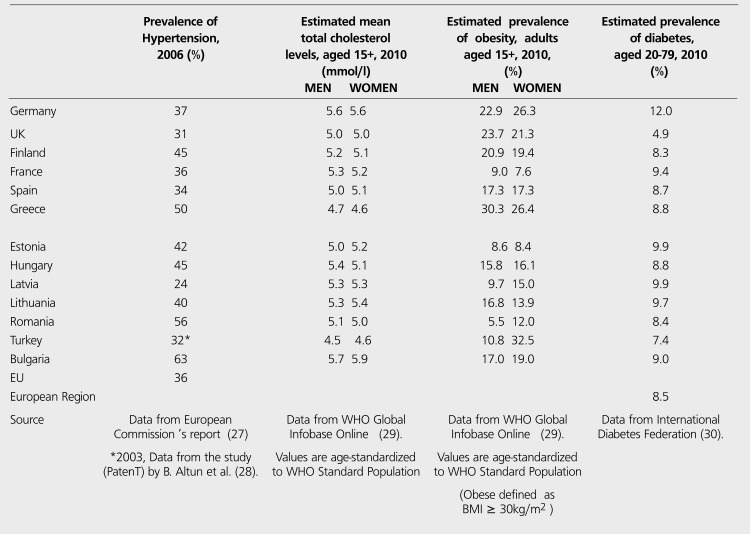
Medical risk factors related to CHD in some European countries

**Table 4:**
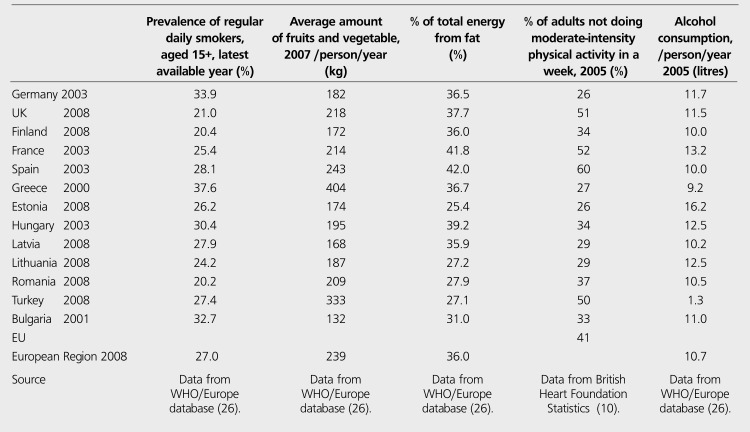
Behavioral risk factors related to CHD in some European countries

**Table 5:**
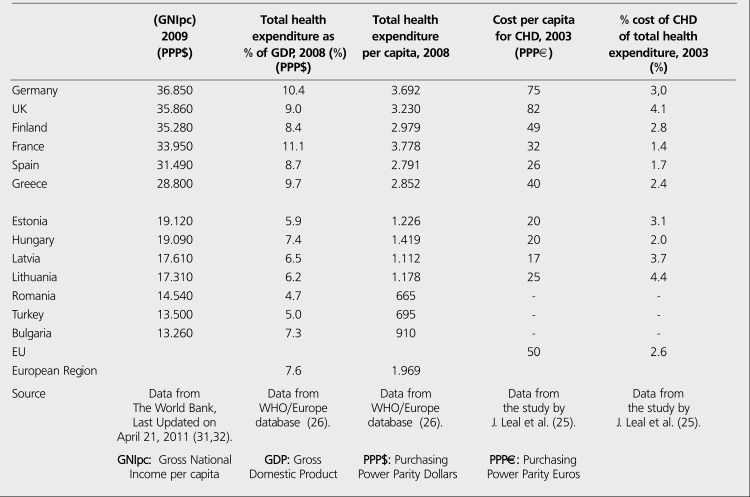
Economic burden of CHD in some European countries

**Table 6:**
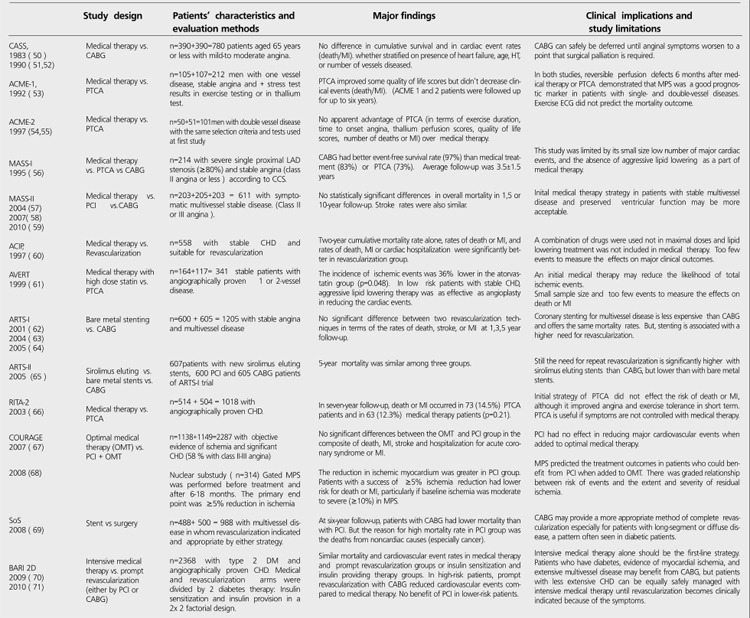
Randomized trials based on treatment strategies in stable patients with CHD

**Table 7:**
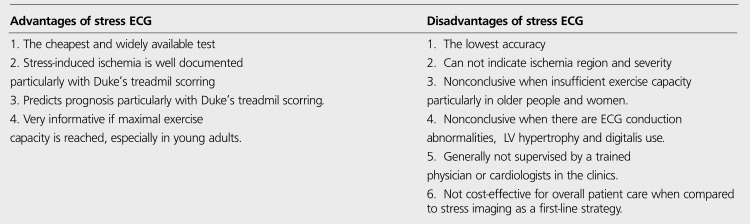
Advantages and disadvantages of Stress ECG in comparison to stress imaging methods (76,81,82)

**Table 8:**
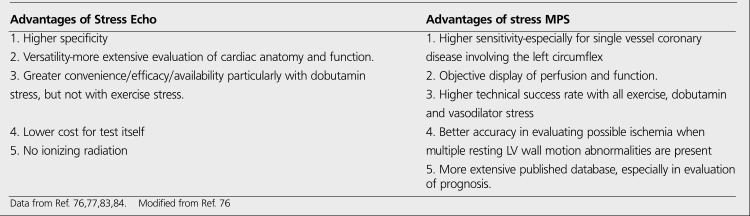
Comparative advantages of stress echocardiography and stress MPS in diagnosis of CHD

**Table 9:**
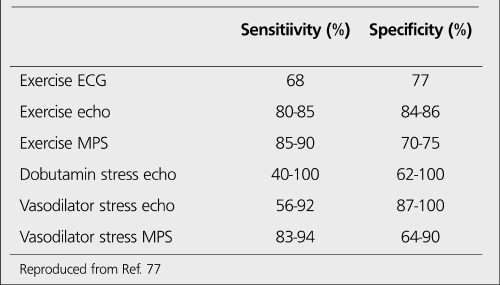
Summary of noninvasive test characteristicsused in the diagnosis of stable angina (77)
